# Calendar

**DOI:** 10.1155/S1463924682000145

**Published:** 1982


					Journal of Automatic Chemistry, Volume 4, Number (January-March 1982), page 50
Calendar
Editor's Note:
Organizers of conferences, seminars, etc.
should send details for inclusion in the
calendar as soon as the relevant infor-
mation is available and not later than three
months before the event.
March 1982
1982 Pittsburgh Conference and Exposi-
tion on Analytical Chemistry and Applied
Spectroscopy
To be held from 8 to 13 March at the
Atlantic City Convention Hall, Atlantic
City, New Jersey, USA.
Contact The Pittsburgh Conference,
437 Donahl Road, Department J-212,
Pittsburgh, PA 15235, USA.
Royal Society of Chemistry: Near Infra-
red Analysis--Today or Tomorrow?
Joint meeting ofthe Analytical Division's
East Anglia Region, Automatic Methods
and Special Techniques Groups. To be
held on 23 March at the University of
East Anglia, Norwich, UK.
Contact Miss P. E. Hutchinson, Royal
Society of Chemistry, Burlington House,
London W1 V OBN.
Royal Society of Chemistry: Annual
Chemical Congress
To be held from 30 March to 2 April at the
University of Aston in Birmingham, UK.
Contact Dr J. F. Gib.on, Royal Society of
Chemistry, Burlington House, London
W1V OBN.
April 1982
Electroanalysis Symposium
Organized by the Electroanalytical
Group and Western Region of the
Analytical Division of the Royal Society
ofChemistry. To be held from 5 to 8 April
at the University of Wales Institute of
Science and Technology (UWIST),
Cardiff, UK.
Contact Dr J. D. R. Thomas, Department
of Chemistry, UWIST, Kin9 Edward VII
Avenue, Cardiff CF1 3NU, UK.
12th Annual Symposium on the Analytical
Chemistry of Pollutants
To be held from 14 to 16 April at the
Free University, Amsterdam, The
Netherlands.
Contact Professor Dr R. W. Frei, Congress
QJJce, 12th Annual Symposium on the
Analytical Chemistry of Pollutants,
Congress Bureau, Vrije Universiteit, PO
Box 7161, 1007 MC Amsterdam, The
Netherlands.
International Congress on Automation in
the Clinical Laboratory
Organized by IUPAC and the Sociedad
Espafiola de Quimica Clinica. To be held
from 19 to 22 April at the Palacio de
Congresso, Barcelona, Spain.
Contact the Secretariat, International
Congress on Automation in the Clinical
Laboratory, Apartado de Correos 543,
Barcelona, Spain.
Analytica '82
To be held from 27 to 30 April in Munich,
FR Germany.
Contact ECL Exhibition Agencies Ltd, 11
Manchester Square, London W1M 5AB.
14th Annual Symposium on Advanced
Analytical Concepts for the Clinical
Laboratory
Sponsored by the American Association
for Clinical Chemistry and Oak Ridge
National Laboratory. To be held on 29
and 30 April at Riverside Motor Lodge,
Gatlinburg, Tennessee.
Contact Carl A. Burtis, Oak Ridge
National Laboratory, PO Box X, Oak
Ridge, Tennessee 37830, USA.
June 1982
'Chemistry Days in Analytical Chemistry'
(Kemistdagar Analytisk Kemi)
Organized by the Analytical Section of
the Swedish Chemical Society. To be held
from 15-18 June in Lurid, Sweden.
Contact the Swedish Chemical Society,
Upplandsgatan 6A, tr, S 11123
Stockholm.
Flow Analysis II
Organized by the Analytical Section of
the Swedish Chemical Society. To be held
from 18 to 21 June at the Hotel Sparta,
Lund, Sweden.
Contact Flow Analysis II, c/o The Swedish
Chemical Society, Upplandsgatan 6A, tr,
S 11123 Stockholm.
International Conference on
Chromatography and Mass Spectrometry
in Biomedical Sciences
Organized by the Italian Group for
Mass Spectrometry and the International
Scientific Center. To be held from 21 to 23
June at Bordighera, Italy.
Contact Dr Alberto Frigerio, c/o Istituto
di Richerche Farmacologiche 'Mario
Negri', Via Eritrea 62, I 20157 Milan,
Italy.
July 1982
Swansea Summer School of Automatic
Chemical Analysis
To be held from 11-16 July at University
College, Swansea, UK.
Contact Professor D. Betteridge, Chemistry
Department, University College, Singleton
Park, Swansea SA2 8PP, UK.
50

				

